# California Serogroup Viruses in a Changing Canadian Arctic: A Review

**DOI:** 10.3390/v15061242

**Published:** 2023-05-25

**Authors:** Jumari Snyman, Louwrens P. Snyman, Kayla J. Buhler, Carol-Anne Villeneuve, Patrick A. Leighton, Emily J. Jenkins, Anil Kumar

**Affiliations:** 1 Department of Biochemistry, Microbiology and Immunology, College of Medicine, University of Saskatchewan, Saskatoon, SK S7N 5E5, Canada; 2 Department of Veterinary Microbiology, Western College of Veterinary Medicine, University of Saskatchewan, Saskatoon, SK S7N 5B4, Canada; 3 Research Group on Epidemiology of Zoonoses and Public Health (GREZOSP), Faculty of Veterinary Medicine, Université de Montréal, Saint-Hyacinthe, QC J2S 2M2, Canada

**Keywords:** *Aedes*, climate change, distribution potential, Jamestown Canyon virus, mosquito-borne, *Orthobunyavirus*, snowshoe hare virus, vector

## Abstract

The Arctic is warming at four times the global rate, changing the diversity, activity and distribution of vectors and associated pathogens. While the Arctic is not often considered a hotbed of vector-borne diseases, Jamestown Canyon virus (JCV) and Snowshoe Hare virus (SSHV) are mosquito-borne zoonotic viruses of the California serogroup endemic to the Canadian North. The viruses are maintained by transovarial transmission in vectors and circulate among vertebrate hosts, both of which are not well characterized in Arctic regions. While most human infections are subclinical or mild, serious cases occur, and both JCV and SSHV have recently been identified as leading causes of arbovirus-associated neurological diseases in North America. Consequently, both viruses are currently recognised as neglected and emerging viruses of public health concern. This review aims to summarise previous findings in the region regarding the enzootic transmission cycle of both viruses. We identify key gaps and approaches needed to critically evaluate, detect, and model the effects of climate change on these uniquely northern viruses. Based on limited data, we predict that (1) these northern adapted viruses will increase their range northwards, but not lose range at their southern limits, (2) undergo more rapid amplification and amplified transmission in endemic regions for longer vector-biting seasons, (3) take advantage of northward shifts of hosts and vectors, and (4) increase bite rates following an increase in the availability of breeding sites, along with phenological synchrony between the reproduction cycle of theorized reservoirs (such as caribou calving) and mosquito emergence.

## 1. Introduction

Unlike other encephalitic vector-borne viruses of medical and veterinary importance, which tend to be associated with tropical climates, viruses in Arctic ecosystems can replicate, transmit and overwinter at extreme temperatures. Their mosquito vectors and reservoir hosts (such as barren ground caribou, which migrate to northern breeding grounds in summer) also display seasonal hyperabundance and phenotypic synchronicity. Research on the two northernmost members of the California serogroup (CSG) viruses— Snowshoe Hare virus (SSHV) and Jamestown Canyon virus (JCV)—was primarily conducted in the 1970s and 80s. Recent reports in people and wildlife in the Canadian North have raised a need to revisit the distribution, ecology, and climate sensitivity of these emerging, neglected viruses. In particular, the application of newer molecular tools is needed; most studies previously focused on La Crosse virus (LACV) and other orthobunyaviruses with more temperate distributions, such that vaccines and antiviral treatments are almost completely lacking for both SSHV and JCV. While the phylogeography, interspecific viral reassortment and the theratogenic effects of these viruses on wild and domestic animals are topics that warrant exploration, it is beyond the scope of this review. Here we take a closer look at the life cycle of these Arctic-adapted viruses. We review and critically evaluate the virus biology, vector status of mosquitoes associated with transmission and highlight the epidemiology of these viruses with regard to host species and human cases, and how all this will be, or already is being, affected by global climate change. This is particularly important in light of recent findings that climate change is occurring at four times the global rate in the Arctic [1], and that climate change will cause increased viral spill over events in a warming Canadian Arctic [2].

## 2. The Viruses

### 2.1. History of SSHV and JCV

The family *Peribunyaviridae* in the order *Bunyavirales* includes globally distributed viruses of medical and veterinary importance in the genera *Orthobunyavirus*, *Herbevirus*, *Pacuvirus* and *Shangavirus* [3]. The *Orthobunyavirus* genus consist of 18 serologically and genetically related mosquito-borne serogroups, including the California serogroup. California serogroup viruses are now recognized as the second-most-common cause of arbovirus-associated neurological disease in North America [4,5]. Viruses in this genus include SSHV, JCV, and LACV, among others, all prevalent throughout North America. These viruses are classified as potentially re-emerging viruses, threatening public health. Snowshoe Hare virus was first isolated in 1958 from the serum of a lethargic Snowshoe Hare (*Lepus americanus*) in Montana, USA [6], with the first human disease documented in the province of Québec, Canada, in 1978 [7]. Jamestown Canyon virus was first isolated in the US from a pool of *Culiseta inornata* mosquitoes collected in Jamestown Canyon near Boulder, Colorado, in 1961 and has recently been identified as an emerging cause of febrile and neuroinvasive disease in Canada and the USA. Although SSHV and JCV are typically asymptomatic, both viruses have been reported in all provinces and territories, including the northern territories in Canada [8,9,10].

### 2.2. Genome Structure and Replication

**Figure 1:**
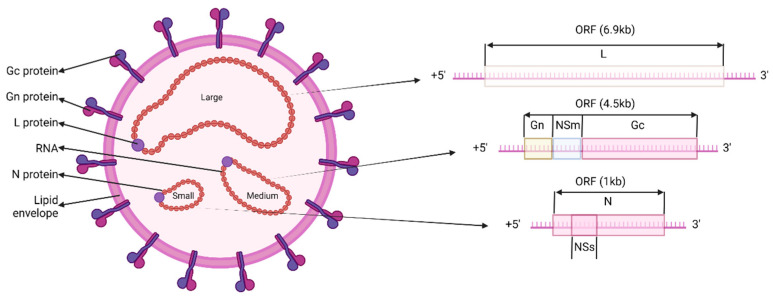
A schematic representation of a Orthobunyvirus virion and the genome organization. The three RNA segments (S: small, M: medium and L: large) are encapsidated by the N (nucleocapsid) protein. For replication to occur, the negative-sense viral genome is converted into positive-sense antigenomes. Adapted from [19]. Created with BioRender.com.

Orthobunyavirus virions are enveloped, spherical or pleomorphic in shape, (80–120 nm) with glycoprotein surface projections (5–18 nm) embedded in a lipid bilayer envelope (about 5 nm) (Figure 1) [3,11]. These viruses have negative-sense, single-stranded, tripartite RNA genomes consisting of three segments designated S—small, M—medium and L—large (Figure 1) [3,12,13]. The S segment encodes two proteins in overlapping reading frames: the nucleoprotein (N) and an intracellular non-structural protein (NS_s_) [12,14,15,16,17,18]. The non-structural protein is a major virulence factor and is not essential for replication [19]. The M segment encodes two surface glycoproteins, termed G_n_ and G_c_, as well as a second non-structural protein, NS_m_ (Figure 1). The two glycoproteins are type I integral membrane proteins that enable viral attachment and entry into the host cell and are considered important determinants of virulence [13,15,20,21]. The NS_m_ non-structural protein comprises three hydrophobic domains (I, III, and V) and two non-hydrophobic domains (II and IV), and is required for virus assembly as demonstrated for Bunyamwera virus (BUNV), although not essential for virus viability [22]. The M segment also determines the viral neutralization phenotype, with G_n_ and G_c_ shown to be the major proteins that elicit neutralizing antibodies against LACV [23]. The L segment encodes the viral RNA-dependent RNA polymerase (RdRp) that catalyses transcription and replication [21,24]. The three genomic RNA segments are encapsidated by the N protein to form ribonucleoprotein complexes that associate with the RdRp and are contained within the lipid envelope of the particle (Figure 1) [19].

**Figure 2:**
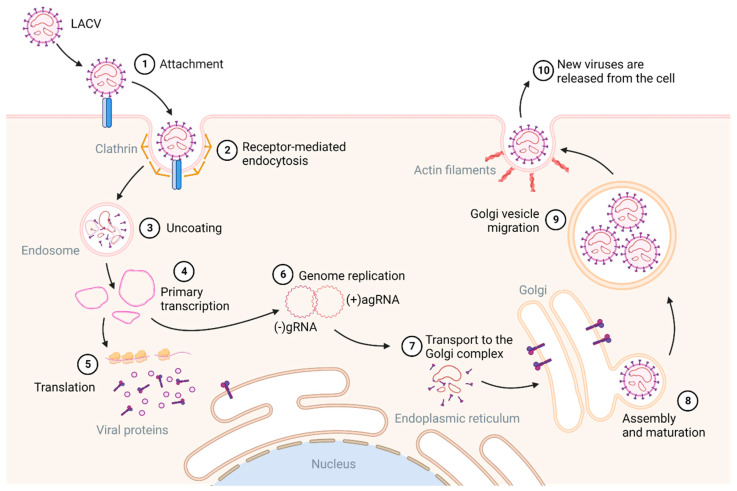
A schematic representation of La Cross virus (LACV) replication. Adapted from [19]. Created with BioRender.com.

Even though JCV and SSHV are the most common CSG viruses found in Canada, they are also among the most neglected CSG viruses [8,25,26]. Few studies have elucidated the replication cycle of these viruses, with most information inferred from studies of BUNV, the Orthobunyavirus prototype, or from the closely related LACV within the CSG viruses. Replication of Orthobunyaviruses starts with the virion attaching via surface glycoproteins and entering the cell through clathrin-mediated endocytosis (CME) (reviewed by [19,27] (Figure 2)). After this process, now called receptor-mediated endocytosis, virion uncoating and the fusion of the viral membrane with the endosomal membrane releases the viral genomes into the cytoplasm. The primary transcription of viral mRNAs, facilitated by RdRp, is initiated, resulting in mRNAs that contain host-derived nucleotides at their 5′ ends [5,19,27,28]. Viral mRNA translation yields the two structural surface glycoproteins G_n_ and G_c_ and the nonstructural protein, NS_m_. The glycoproteins enter the endoplasmic reticulum (ER) from where they localize to the Golgi complex, where replication occurs. For replication to occur, the negative-sense viral genome is converted into positive-sense antigenomes, resulting in ribonucleoproteins (Figure 1 and Figure 2). Virus particles bud into the Golgi membrane-derived vesicles from here they are trafficked to the cell surface. Virions are released from the cell via exocytosis, and the glycoproteins undergo a final maturation step upon exit (Figure 2).

The receptor usage of bunyaviruses is still incompletely understood. Recently, studies have focused on identifying such receptors, although none specifically for SSHV or JCV have been identified. Studies on host cell receptors primarily focused on phleboviruses (order *Bunyavirales*, family: *Phenuiviridae*) and to a lesser extent, LACV. Dendritic cell-specific intercellular adhesion molecule-3-grabbing non-integrin (DC-SIGN) and liver-specific SIGN (L-SIGN) bind carbohydrate structures present on viral glycoproteins and function as attachment factors for several enveloped arboviruses, including Germiston virus, an orthobunyavirus (DC-SIGN only) [29], phlebo- [29,30] and alphaviruses [31]. Hofmann et al. [32] demonstrated DC-SIGN, but not L-SIGN, as a potential receptor for LACV entry—the first data for CSG viruses. This study also demonstrated that pseudotyped virus-bearing LACV G_n_/G_c_ has overlapping but not identical tropisms for human and animal cells compared to phleboviruses, suggesting that these genera have different receptor requirements for host cell entry. Heparin sulfate proteoglycan is a glycoprotein expressed on the cell surface of all animals that acts as an attachment receptor for various arboviruses [33,34], including two orthobunyaviruses (Akabane virus and Schmallenberg virus). However, further studies are needed to determine if this is an important attachment factor for the CSG viruses, contributing to the emerging field of active molecular biology research on bunyaviruses.

Earlier studies have demonstrated that LACV, SSHV, and to a lesser extent, JCV, are sensitive to lysosomotropic agents, indicating that virions enter cells by receptor-mediated endocytosis and not by direct fusion at the plasma membrane [35]. Elsewhere [36], CME has been extensively demonstrated for LACV in various cell lines, including primary neuronal cultures. This study also demonstrated that both SSHV and JCV use CME to enter cells [36], and that macropinocytosis and caveolar endocytosis, both established routes of viral entry, are not critical for the cellular entry of LACV. Successful LACV infection depends on Rab5, a key regulator of endocytic transport to early endosomes, which plays an important regulatory role in early endosomes, but not on Rab7, which is associated with late endosomes. Clathrin, or receptor-mediated endocytosis, is an entry mechanism used by many viruses, including encephalitic viruses such as West Nile virus [37] and Japanese encephalitis virus [38], and is a possible pathway for neuroinvasion for CSG viruses. For LACV, amino acids 860–1442 of the G_c_ glycoprotein have been identified as the principal determinant of virus fusion and cell entry [39]. Additionally, a small segment within this region was predicted to have structural homology with the fusion protein (E1) of Sindbis virus [39] that is important in alphavirus cell entry and fusion [40]. The N protein is the main protein produced in infected cells and interacts with both genomic and antigenomic RNA to play a vital role in viral replication. Ogg and Patterson [17] demonstrated that, similar to BUNV, the 5′ end of JCV contains the viral RNA region responsible for binding to the nucleocapsid.

### 2.3. Pathogenesis, Human Infection, and Host Responses

Most of the current understanding of the transmission, dissemination, and pathogenesis of CSG viruses in mammals is based on studies with LACV in murine models, with a few studies in non-human primates as models for vaccine development. The infection of CSG viruses is initiated by the bite of an infected mosquito. After this, the virus replicates near the site of entry in the striated muscle, lymph nodes, and/or spleen [5]. The virus then disseminates to the blood, probably through the lymphatics system, resulting in a serum viremia followed by crossing of the blood–brain barrier (BBB) to the central nervous system (CNS). Once in the CNS, the virus targets the cortical and basal ganglia neurons and possibly astrocytes [41], leading to inflammatory lesions with monocytic infiltration and lymphocytic perivascular cuffing that results in encephalitis. Encephalitis has been associated with a high serum viremia presumably because a high viral titre enhances the entry of the virus across the BBB. The exact entry route of LACV into the CNS, and overcoming the BBB, is not clearly defined. A possible route via the olfactory neurons has been reported [42,43]. LACV infection of the CNS is age-related and juvenile mammals seem to be more susceptible than adults. Previous case reports of SSHV disease in Canada have described encephalitis predominantly affecting young children, although cases affecting adults have been described [4,7,9,26,44,45,46]. In contrast, for JCV, neuroinvasive disease has been reported in children, young adults, and older adults [46,47,48].

Despite the literature supporting a high risk of exposure to SSHV and JCV across Canada, clinical disease is infrequently documented. Most cases of CSG viruses were reported between 1978 and 1989, with at least 1 symptomatic infection reported annually in Canada and a total of over 20 cases during that time period [9]. Of the 20 cases, SSHV was mostly implicated as the aetiology [9]. The next reported human infection of either SSHV or JCV in Canada was in 2006 [4], once serology for CSG viruses became available in 2005, leading to an increase in the recognition of clinical cases in Canada [8]. The low case rate for CSG viruses could be attributed to subclinical or asymptomatic infections, along with under-diagnosis and under-reporting. Testing is not often requested due to a lack of awareness among health care providers regarding CSG virus epidemiology and clinical manifestations [25,49,50], and the virus is not notifiable to national public health authorities in Canada. In 2019, the National Microbiology Laboratory (NML) in Winnipeg, Manitoba, reported 14 JCV cases and 1 SSHV case, with 4 unidentified CSG virus cases [46]. The National Institute of Public Health of Québec (INSPQ) reported 15 JCV and 1 SSHV cases in 2019, 3 JCV and 3 SSHV cases in 2020 and 7 JCV and 6 SSHV cases in 2021. Clinical manifestations of SSHV and JCV include headache, fever, dizziness and vomiting, while photophobia, respiratory distress and rashes have also been documented. Viral encephalitis is characterized by fever, headache and altered mental status ranging from confusion to coma, with or without additional signs of brain dysfunction, and even death [4,26,48,50]. There are few studies that estimate human exposure in Canada, with most focusing on the maritime provinces of New Brunswick and Nova Scotia. Here, PRNT results indicated that most positive results are due to exposure to JCV [25,51,52].

### 2.4. Animal Models and Therapeutics Developments

Determining the pathogenesis of these viruses in animals and cell culture models could help explain the difference in disease outcomes observed in humans. The disease outcome in mouse models closely resembles human disease, and infection in weanling mice allows for age-dependent susceptibility studies [5,53]. Studies have demonstrated the difference in neuropathogenesis between weanling and adult mice after infection with LACV and SSHV [5,53]. The age-dependent effect was attributed to type I IFN responses, which were significantly stronger in adult mice compared to weanling mice [53]. Evans et al. [5] demonstrated that LACV and SSHV induce neurological disease after intraperitoneal and intranasal inoculation; however, JCV induced disease only after intranasal inoculation. Similar results were observed by Bennett et al. [54], who reported low levels of neuroinvasiveness following the intraperitoneal inoculation of weanling mice with JCV, although neurovirulence was observed between different clones of the virus.

Mouse models are also readily used for targeted antiviral development and vaccine efficacy studies. As emerging viruses, the development of vaccines or antiviral treatments against SSHV and JCV could be beneficial as currently only supportive care is possible for clinical cases and no vaccine has been approved for human use to date. A live attenuated orthobunyavirus vaccine candidate has been developed for JCV that successfully induced neutralizing antibodies in people and protected monkeys against developing viraemia after JCV infection [55]. As for antiviral therapy, limited studies have examined compounds against SSHV and JCV. Favipiravir (FPV/ T-705) showed an inhibitory effect against JCV replication in African green monkey kidney cells (VERO) as well as neuro-2a cells, and prolonged survival rate and delayed the onset of neurologic disease in mice inoculated intracerebrally with JCV [56]. Favipiravir has also demonstrated protection, both in vitro and in vivo, against other encephalitic viruses such as West Nile virus [57] and Western equine encephalomyelitis virus [58], demonstrating the broad promise of FPV against arboviral encephalitides. Kato et al. [56] also reported on the inhibitory effect of ribavirin on JCV replication in vitro, although in vivo results were not significant. Ribavirin was also ineffective in treating LACV infection in vivo [59] and showed no antiviral activity against other orthobunyaviruses such as Oropouche, Caraparu, Guama, Guaroa, or Tacaiuma viruses in mouse models [60]. Other antiviral compounds inhibiting LACV replication include valinomycin, lapachol, and lagistase, with valinomycin exhibiting broad activities against various viruses [61], and should be included in SSHV and JCV therapeutic studies.

## 3. The Vectors

### 3.1. Distribution and Abundance of Potential Vector Species

Mosquitoes are the primary vectors of CSG viruses in North America, even though mechanical transmission by biting flies such as *Tabanidae* may also contribute to their transmission cycle. To date, approximately 82 species of mosquitoes are thought to occur in Canada [62] (https://www.mosquitocatalog.org/—accessed on 15 January 2023). Jamestown Canyon virus and SSHV have been isolated from several mosquito species across a wide taxonomic range [63,64]. The local detection of virus, or viral RNA, in field-trapped mosquitoes is an indication that the virus is circulating among vertebrate hosts in the region; however, this cannot be considered evidence of vector competency. As an example, in the USA, JCV was isolated from field-caught *Anopheles quadrimaculatus* [65]. However, Dieme *et al.* [66] experimentally determined that this species is not a competent vector for two strains of JCV naturally circulating in the northeastern USA.

Jamestown Canyon virus has been isolated from more than 25 mosquito species across temperate North America [63,67], including *Aedes triseriatus*, *Ae. provocans*, *Ae. squamiger*, *Ae. dorsalis*, *Ae. canadensis*, *Anopheles punctipennis* and *Coquillettidia perturbans*—all common species found in Canada. Regionally, JCV has been isolated once from a pool of *Aedes communis* (Alberta), once from a pool of *Ae. abserratus*/*punctor* (Newfoundland and Labrador) and twice from mixed *Aedes* pools (Alberta, Quebec) [64,68,69]. The transmission of JCV (i.e., vector competence) has been demonstrated for only a few species: *Ae. canadensis*, *Ae. provocans*, *Cs. inornata* and *Cq. perturbans* found throughout Canada, and *An. punctipennis* in southern regions [62,70].

**Figure 3:**
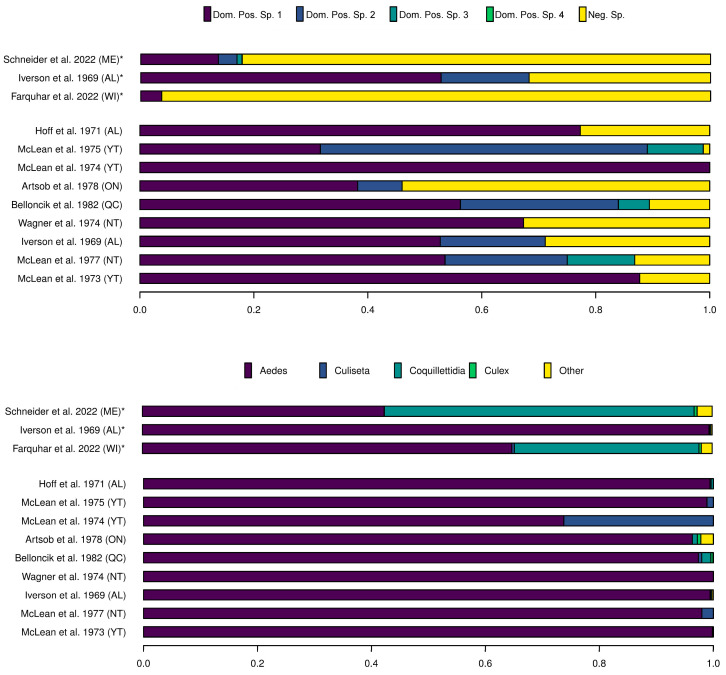
(**Top**) Proportions of dominant mosquitoes species from which Jamestown Canyon virus (denoted with a *) and Snowshoe Hare virus was isolated (Dom. Pos. Sp. 1–4) compared to the collective proportion of species that tested negative for virus (Neg. Sp.); (**Bottom**) showing that JCV is more vector specific whereas SSHV was found in most common mosquito species. Data taken from studies cited on y axis. ME: Maine (USA); AL: Alberta; WI: Wisconsin (USA): YT: Yukon Territory; ON: Ontario; QC: Québec; NT: Northwest Territories.

*Aedes provocans* is present in all Canadian provinces except Yukon, Newfoundland, and Labrador [62], and was the sole species from which JCV was isolated during an outbreak in Wisconsin (USA) [71]. The outbreak virus was isolated from several pools of adults over two years, while another viral isolate was detected in larvae. Interestingly, *Ae. provocans* was not the most abundant species, accounting for approximately only 3% of the total sample (Figure 3) [71]. In fact, the most frequently sampled species was *Cq. perturbans* (~38%), followed by *Ae. canadensis* (~14%), with both species thought to be important vectors of JCV. Similarly, JCV was not isolated from *Cq. perturbans* (accounting for nearly 50% of the sampled and screened mosquitoes) in a study in Maine (USA), but was instead isolated from *Ae. cantator*, *Ae. provocans*, *Ae. sollicitans* and *Uranotaenia sapphirina* [67]. The vector dynamics of JCV are thus not clearly understood and regional surveillance as well as vector competence studies are clearly warranted.

In contrast with JCV, which appears to have high vector specificity, SSHV is commonly isolated from the most abundant or dominant species of field-caught mosquitoes in the Canadian North, which are usually *Aedes* spp. (Figure 3) [68,69,72,73,74,75,76,77]. As an example, SSHV was isolated from five mosquito species (*Aedes canadensis*, *Ae. cinereus*, *Ae. communis*, *Ae. hexodontus* and *Cs. inornata*) and was the most abundant, generally comprising more than half of the sampled mosquitoes. SSHV was only isolated from *Cs. inornata* when it accounted for a larger proportion of the sample [75].

Furthermore, SSHV is often considered vectored by “non-*Culex*” mosquitoes; however, a wide range of mosquito species are potential vectors of SSHV, and it may be that the current distribution of SSHV merely coincides with that of *Aedes* spp. and *Cs. inornata* and not *Culex* spp. From an evolutionary perspective, *Culiseta* is more closely related to *Culex* than *Aedes* [78], suggesting that further work is needed to determine if SSHV can be vectored by *Culex* mosquitoes. The distribution of SSHV may thus not be limited to the distribution of its currently recognized vectors.

Our understanding of the spatiotemporal distribution of CSG viruses in mosquitoes in the Arctic and Subarctic is mostly based on surveillance conducted during the 1970s and 80s [68,69,72,73,74,75,76,77]. However, vectoral capacity might differ through space and time. As an example, the vector status of *Ae. stimulans* appears to vary regionally. *Aedes stimulans* in Indiana (USA) transmits JCV vertically and horizontally, and has an apparent preference for white-tailed deer, which become viraemic for JCV [79]. There are several studies north of Indiana, however, where JCV was not isolated from *Ae. stimulans*, despite the species being proportionally well represented and sympatric with white-tailed deer reservoirs [67,68,71,73,80]. Therefore, updated and regionally relevant richness and abundance studies and surveillance to detect distribution shifts in space or time, both critical components in the maintenance cycles of arboviruses, are needed.

Although we do not have good regional data on arbovirus transmission, understanding the phenology and biology of mosquitoes can help generate hypotheses and guide surveillance efforts. In the Yukon, the potential vectors *Ae. cinereus* and *Cs. inornata* evidently emerge during spring thaw when temperatures remain low [75]; however, *Ae. canadensis* and *Ae. communis* populations quickly become dominant when temperatures rise above 26.7 °C [76]. In contrast, Wood [62] states that *Ae. cinereus* overwinter as eggs and are perhaps of one of the last species to hatch in spring. The emergence time of *Ae. cinereus* thus requires clarification. It is also worth noting that while both *Ae. canadensis* and *Ae. cinereus* are seemingly univoltine in the Yukon, bivoltine and mulitivoltine populations exist where conditions are suitable, an important phenology considering virus transmission and climate change [62].

Moving eastward, *Ae. hexodontus* gradually become more abundant and are present in high numbers alongside *Ae. communis* in the Northwest Territories [77]. Further east, in Nunavut, *Ae. hexodontus* and *Ae. nigripes* completely replace *Ae. communis* as the dominant species [74]. *Aedes hexodontus* is the species responsible for the formidable reputation the Arctic has for mosquitoes [62]. The eggs of this species hatch at around 0 °C, with larvae displaying a high minimum temperature threshold, getting a head start on sympatric species [62].

East of Hudson Bay, *Ae. punctor* is seemingly the most frequently trapped species and accounted for most of the samples in studies from Québec, Newfoundland and Labrador [69,72]. Unlike the morphologically similar *Ae. hexodontus*, which prefers open country, *Ae. punctor* is mostly confined to the boreal forest. The eggs of *Ae. punctor* also hatch in early spring when ice is still melting, and are among the first species to pupate [62].

*Culiseta inornata* is found across western and southern Canada and is strongly associated with the prairies [62]. *Culiseta inornata* overwinter as adults in the burrows of vertebrates and are therefore among the first adult mosquitoes present after the winter at low temperatures and have even been observed feeding on humans at temperatures below 0 °C [62,76]. Coupled with the known replication of CSG viruses in the salivary glands of *Cs. inornata* at temperatures as low as 5 °C, the virus may well overwinter in *Cs. inornata* adults [76].

The spatial and/or temporal distribution, species succession, voltinism, regional vectoral capacity, and overwintering of mosquitoes is thus fragmented, not well understood and outdated. These life histories might further be altered by climate change and in turn alter the epidemiological dynamics of CSG viruses.

### 3.2. Transovarial Transmission and Temperature Thresholds

Overwintering via transovarial transmission is common for Orthobunyaviruses and likely plays an important role in the enzootic maintenance of both JCV and SSHV [81] in the Canadian North [8]. The transovarial transmission of JCV has been demonstrated in the USA for several species of mosquitoes that also occur in Canada. The virus has been isolated from adult *Ae. triseriatus* reared from field-collected eggs in Ohio [82], from adult *Ae. provocans* reared from larvae in Wisconsin [71], from male *Ae. stimulans* specimens in Indiana [79] as well as from immature *Ae. abserratus* and *Ae. cataphylla* [81]. JCV can infect *Culiseta inornata* both horizontally (via hosts) and vertically (generationally) [83].

While the vertical transmission of JCV is clearly a possible overwintering mechanism, transovarial transmission may be less efficient at cold temperatures. In a study in California, reducing temperature from 20 °C to 10 °C while incubating *Ae. squamiger* larvae reduced the infection rate of the resulting adult female mosquitoes [83]. This sensitivity of JCV to temperature highlights the need to determine the thermal thresholds of JCV below which viral replication and/or survival are affected. JCV could infect *Aedes cataphylla* horizontally but not vertically when larvae were incubated at 5 °C for 10 days, followed by 10 °C until emergence [83]. These low temperatures likely imitate the natural conditions experienced by *Ae. cataphylla* at high altitudes. Since the distribution range of *Ae. cataphylla* reaches the western Arctic and the species readily feeds on humans—this has relevance for the Canadian Arctic, and warmer temperatures may well enhance the transovarial transmission of JCV.

Transovarial transmission is likely also important for SSHV, which has been isolated in Canada from wild-caught *Aedes* immatures in the Yukon [84], as well as from adult *Ae. implicatus* and *Ae. communis* adults reared from wild-caught larvae in Saskatchewan and Québec, respectively [72,85]. Snowshoe Hare virus is transmitted by both vertical and venereal routes in *Ae. triseriatus* and *Cs. inornata* inoculated by intrathoracic injection [86]. Temperature thresholds are better characterized for SSHV compared to JCV, with viral survival and infectivity varying with infective dose, vector species, and incubation length. For example, wild-caught *Cs. inornata* intrathoracically injected with 30 mouse LD_50_ transmitted SSHV to mice after 17 days incubating at 26.7 °C; the replicating virus was isolated from the salivary glands of *Cs. inornata* injected with only 0.03 mouse LD_50_ after 46 days of incubating at 12.8 °C, but no infectivity was detected after 46 days at 4.4 °C with an infective dose less than 3 mouse LD_50_ [75]. In contrast, SSHV maintained transmission in *Ae. cinereus* at lower temperatures, with wild-caught *Ae. cinereus* readily transmitting SSHV to mice after the intrathoracic injection of at least 3 mouse LD_50_ after 15 days incubating at 12.8 °C. At 4.4 °C, the replicating virus could be isolated from *Ae. cinereus* salivary glands after 57 days [75]. Similarly, the replicating virus could be isolated from the salivary glands of wild-caught *Ae. canadensis* incubating at 4.4 °C after 17 days [76]. Snowshoe Hare virus is thus capable of replicating in vectors at low temperatures for an extended period, providing an additional route for overwintering.

Cool temperatures also restrict the distribution potential of introduced vectors and their viruses. Although not yet broadly established in Canada, the principal vector for many important vector-borne pathogens, such as yellow fever, dengue and Zika, *Aedes aegypti*, is seemingly also a competent vector of SSHV [86]. Even though laboratory colonies of the species could not be maintained at 10 °C [76], SSHV was transmitted via bites after three weeks at 26.7 °C and four weeks at 12.8 °C [86]. Less than half of the mosquitoes infected at feeding transmitted the virus, reaffirming the limiting role temperature plays in transmission [86]. Another invasive species, *Aedes albopictus*, is also now regularly collected in Eastern Canada [87]. Since both species have demonstrated vectoral capacity for a wide range of viruses (including CSG viruses) and exhibit anthropophilic behaviour, monitoring their distribution in a warming future is important.

Further surveillance is needed to update current understanding of vector distribution patterns in Canada, as is experimental work to better determine the thermal limits of both viruses and vectors. Our comprehension of mosquito host range and preferences in the Canadian North is based on only a few relevant reports [79,88,89]. Since heteroxenous lifecycles of arboviruses can be complex, multidisciplinary approaches are needed to assess the mosquito vectorial capacity, vector competence, host preference, and spatiotemporal distribution.

## 4. Mammalian Hosts

Under present and future climate warming scenarios, both SSHV and JCV are predicted to undergo northward range expansion in Subarctic and Arctic regions, where a wide range of mammals already have evidence of exposure to these viruses [90]. As infection with these viruses typically results in transient viremia, most of the published literature from Canada has focused on antibody detection in humans and animals using a competitive ELISA and/or a plaque reduction neutralization test (PRNT) [51,91]. This has revealed a wide range of hosts that can become infected, including humans, lagomorphs, rodents, foxes, polar bears, domesticated livestock (horses, cows, and sheep), and multiple cervids [51,76,91,92,93]. However, there are relatively few mammalian hosts that are thought to be competent reservoirs for the viruses: small mammals (rodents and lagomorphs) for SSHV and wild cervids for JCV [94,95,96]. These animals play a significant role in the enzootic transmission cycle of CSG viruses in temperate regions of Canada and the United States [8]. Other hosts that are exposed to CSG viruses are not thought to contribute significantly to further transmission, though they may be sentinels, indicating the presence of CSG viruses in ecosystems and serving as predictors for human health risk and/or range expansion [97,98].

**Figure 4:**
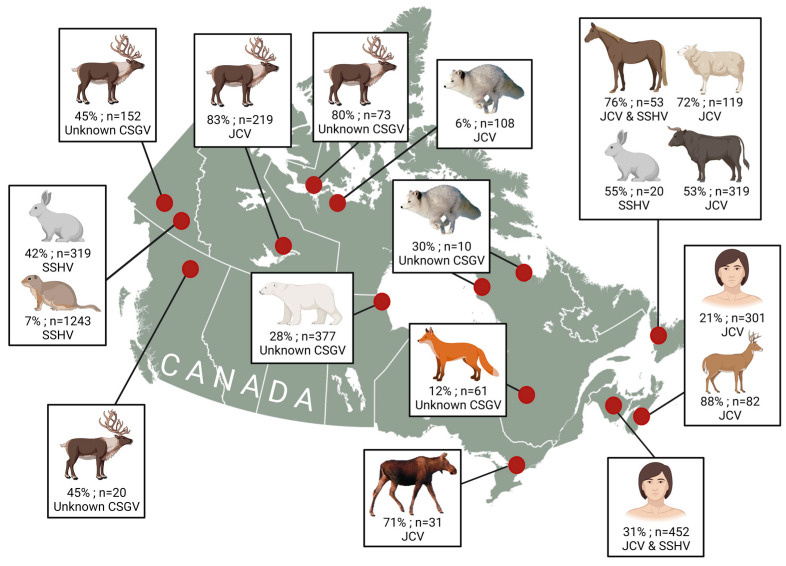
Serosurveys of California seogroup viruses (CSGV) across Canada. When available, the identity of specific CSG viruses, responsible for infection, identified with neutralization tests (PRNT), have been listed under the overall seroprevalence. Human serosurveys have been completed in New Brunswick [25] and Nova Scotia [51]. Serosurveys in animals across Canada includes snowshoe hares, bovines, sheep, horses [76,93], ground squirrels [76], white-tailed deer [51], moose [94], polar bears, Arctic fox, red fox, and caribou [91]. Locations indicate the province/territory where sampling was completed, bearing in mind that some animals undergo long distance movements across borders. Created with BioRender.com.

As expected, JCV is most often found in larger animals, while SSHV is commonly found in small mammals across Canada (Figure 4) [93]. This pattern appears to remain consistent in Arctic regions (Figure 4) [76,91]. Though many species of mosquitoes can carry both SSHV and JCV, there may be differences in vector feeding preferences for some competent vectors, which may contribute to the differences in exposure that are observed in different taxa [99]. Caribou appear to play an important role in the maintenance of JCV in northern ecosystems, as they are highly exposed and are the most common cervid north of the treeline [91]. This is consistent with a previous study that identified white-tailed deer as amplifying hosts for JCV in the United States [100]. In addition, high exposure in lagomorphs from northern provinces and territories suggests that they may be amplifying hosts for SSHV, though experimental infection studies are lacking for northern species [101].

It is difficult to fully understand the transmission dynamics of CSG viruses in the north, as there are few long-term studies in Arctic wildlife. However, one study examined exposure to CSG viruses during three time periods (1986–1989, 1995–1998, 2015–2017) for polar bears from western Hudson Bay and found a significant association between summer air temperatures and antibody prevalence in bears [91], suggesting that exposure to CSG viruses has already and will continue to increase for Arctic wildlife as the climate warms [102]. Bears located near Churchill along the coastline also experienced less exposure to CSG viruses during this study, likely due to the dampening effect of ocean wind on mosquito activity [91,93]. Though bears may serve as sentinels for climate-driven change in the Arctic, it is unknown if they are amplifying hosts for CSG viruses.

Caribou, which are likely to be important maintenance hosts for JCV in the North, exhibit unique behaviours among cervids in Canada. These animals are generally classified as sedentary or migratory, with sedentary caribou (some woodland ecotypes) locally dispersing and migratory caribou moving in large herds to alpine habitats (mountain ecotype) or tundra habitats (migratory tundra ecotype) during the calving season [103]. Depending on the scale of these migrations, caribou may or may not be good sentinels for local risk of CSG viruses, as those that undergo large dispersals could provide a poor signal for CSG virus presence at sampling sites. For example, positive animals in the migratory tundra ecotype may only reflect the presence of CSG viruses around their calving grounds in the Arctic, corresponding with their location during periods of insect activity [91]. Regardless, the movement of caribou herds into tundra ecosystems provides opportunities for the dissemination of JCV, especially as insect activity overlaps with calving and postpartum, when herds are most vulnerable [104]. The reservoir potential of *Rangifer* species, including caribou and reindeer, appears likely, as reindeer are capable of developing viremia for five days following subcutaneous inoculation with JCV and appear to develop minimal health effects following infection [105].

Northern rodents and lagomorphs that are suspected to be involved with SSHV maintenance uniquely exhibit cyclical population irruptions in the Arctic. For example, snowshoe hares have a wide distribution across Canada and undergo 9 to 11 year population cycles in boreal forests [106]. In addition, lemmings and Arctic hares exhibit three to four year cycles in tundra regions [107,108]. Though cyclic and noncyclic hare and rodent populations are probably involved with the maintenance of SSHV in the North, population irruptions with cyclic species provide prime opportunities for the dissemination of SSHV. A population peak that is accompanied by warmer summer temperatures would likely increase the density of amplifying hosts and mosquitoes, creating ideal scenarios for transmission. In addition, these animals have small home ranges, making them important sentinels for local SSHV risk for humans around northern communities [109]. Future studies could focus on serosurveys of these rodent and lagomorph species to identify whether SSHV and other viruses are present across Arctic ecosystems, along with experimental infection studies to verify if they are suitable amplifying hosts.

There is no information available for the duration of antibody production in northern species, limiting the interpretation of results from long-lived animals that may be exposed over multiple summers [91]. However, sampling animals with short lifespans or sampling the young of the year may provide a clear picture of seroprevalence during specific time-periods.

## 5. Climate Change

Over the past 50 years, increased global connectivity and anthropogenic climate change in the Arctic have altered the distribution and abundance of pathogens, contributing to the frequency of emerging infectious disease outbreaks [110]. In particular, the western Canadian Arctic has experienced a warming trend of ~2.5 °C, compared to the Canadian national average of 1.9 °C, since monitoring began in 1948 [111]. The recent estimates suggest that the Arctic is warming at four times the global rate [1]. Northward advancement of the treeline and a 50–60% increase in Arctic precipitation over the past 20 years have also been observed in tundra ecosystems [112,113]. Climate models project that these patterns will continue in the future, with increased warming in northern regions and higher rates of warming predicted in the winter versus the summer [102]. Similarly, climate models predict higher rates of precipitation, with roughly a 7–24% increase depending on low- and high-emission scenarios, and a shift from snow to rain in the spring and fall seasons [102]. All these changes may break down barriers for the emergence of arboviruses by altering the population dynamics of viruses, vectors, and wildlife hosts.

As the CSG viruses do not have an environmental stage, the most climate-sensitive part of the life cycle is in the mosquito poikilothermic hosts, rather than in the mammalian endothermic hosts. In general, most vector-borne viruses have higher rates of development in vectors (i.e., shorter extrinsic incubation periods) under warmer temperatures, suggesting that there will be an increased amplification of the circulating virus in currently endemic regions (which includes most of Canada, including the northernmost communities). This is at least the case expected for West Nile virus under different climate change scenarios [114]. Temperatures are not likely to warm above the viral survival limits (especially in the Arctic), since mosquitoes are mobile hosts and will seek shelter before exceeding their survival threshold. Likewise, these viruses are not likely to disappear at their southern distributional limits within Canada since they are both currently present well into the United States. While there is limited information, empirical evidence indicates that warming temperatures will increase both the overwinter survival of viruses in mosquitoes, and perhaps most importantly, the success of transovarial transmission of viruses within mosquitoes. In addition, the infectivity of SSHV is higher in mosquitoes incubated at higher temperatures. If, as it appears, SSHV is more capable of using different vector species, it will likely be less affected by climate-driven changes in vector diversity than JCV. In short, warmer conditions will likely lead to increased viral survival, development rates, and transmission in endemic regions, without losing viral establishment in southern regions, and possibly increasing their northern distributional limits along with their hosts and vectors.

The overall effect of climate warming on mosquito vectors in the Canadian Arctic is likely to be positive and substantial, in the form of shifts in life-history traits of arthropods, including the survival, fecundity, development time, host-seeking behaviours and phenology of interspecies interactions [115,116]. Warmer winter temperatures and thicker snowpacks may favour the survival of adult mosquitoes in dens of vertebrate hosts. Decreasing winter mortality due to a decrease in the number of winter days with extremely low temperatures is likely to cause a northward shift in the current distribution of some *Aedes* species [117]. Warming temperatures could result in an earlier hatching of mosquitoes and increase their activity period in the summer [118]. With a longer activity period, the presence and proportion of infected female mosquitoes will increase, and the transmission risk of arboviruses might be higher [119]. Warmer water temperatures in summer will enhance the survival of immature mosquitoes in biofilms and enhance the development of larger and more fecund adults [120]. Warming summer temperatures could decrease egg retention in female mosquitoes, especially *Ae. impiger* [121], making oviposition faster, and shorten the interval between successive gonotrophic cycles [118]. This will also shorten the development time required for immature mosquitoes, thus reducing their exposure to aquatic predators, and increasing survival and the number of generations that can be completed each summer. This short development rate is likely to bring mosquitoes into phenological synchrony with caribou [104]; however, it is also possible that the earlier emergence of adult mosquitos in spring as a result of warming may create a disconnect with migrating caribou, who may be driven more by photoperiod than temperature. In general, longer active seasons will alter the host/vector dynamic by increasing the time available for host harassment, thus providing more opportunities for vector-borne disease transmission [115]. Finally, with regard to mosquito species currently of importance to CSG virus transmission in the Canadian North, *Ae. cinereus* and *Cs. inornata* will likely emerge earlier each spring [75], while *Ae. canadensis* and *Ae. communis* populations will likely continue to dominate in warmer summer temperatures [76]. Both *Ae. canadensis* and *Ae. cinereus* will likely switch from univoltine patterns to bivoltine and even mulitivoltine populations as conditions become more suitable [62]. *Anopheles punctipennis*—currently a southern vector for JCV—may move further north and *Aedes provocans* may be able to colonize regions where it is currently absent. Invasive species such as *Ae. aegypti* and *Ae. albopictus* are competent for JCV and SSHV and may well be able to newly establish further north in Canada. While interspecific competition outcomes and vector competency are too complex to predict, warmer conditions will lead to earlier and longer seasons for mosquito activity, faster development, an increased number of generations of mosquitoes per season, an increased overwinter survival of eggs and adults, and an overall higher abundance, activity, and diversity of mosquitoes competent to serve as vectors for JCV and SSHV in Canada.

In comparison with temperature, changes in precipitation and regional hydrology are also extremely important for mosquito populations, but substantially more difficult to predict on a meaningful scale. While, intuitively, wetter conditions should favour mosquito abundance and reproduction, there may be checks and balances. Overall, precipitation in the Canadian North will increase [122], and likely an increase in standing water in summer will increase available breeding habitat, but melting permafrost will reduce suitable habitat for Arctic mosquito species [104,115,118]. Since their eggs must be submerged over an extensive period to be able to hatch, the permafrost ensures that soil is saturated with water and that temporary waterbeds are flooded [118,123]. Melting of the permafrost may lead to dryer soil, reducing the number of eggs hatching and leading to a population decline in some Arctic species [124,125], especially in competition with drought-adapted southern species [126]. Therefore, the direction of the overall effect of changing moisture conditions on mosquito populations is likely positive, but probably highly regionally variable.

The effects of climate change on mammalian reservoir hosts for CSG viruses are largely unknown. Indeed, further work is needed to determine the actual reservoir hosts in Subarctic and Arctic regions of Canada, hampered by the challenges of transient viremia and access to samples from free-ranging wildlife in remote regions. In general, rodents and hares are the likely reservoir for SSHV, and cervids (predominantly caribou above 60° N in Canada) are the host for JCV. Climate, especially precipitation, is strongly linked to rodent abundance, often with vegetation growth as the intermediary variable [127]. Therefore, increased precipitation could lead to higher rodent abundance overall, but greater variability could drive an increased magnitude of peaks and troughs in rodent populations that might destabilize transmission of CSG viruses. Caribou populations in northern Canada are declining; based on the Species at Risk Act and the Committee on the Status of Endangered Wildlife in Canada (COSEWIC) https://www.canada.ca/en/environment-climate-change/services/species-risk-education-centre/caribou.html—accessed on 15 January 2023), boreal and barren ground caribou are listed as threatened, and Peary caribou and Dolphin-Union caribou are listed as endangered. Declining caribou populations may therefore reduce suitable reservoirs for JCV; however, white-tailed deer (*Odocoileus virginianus*) along with other cervids also appear to serve as competent reservoirs for JCV and are already moving north in Canada, largely in response to climate and habitat alteration [128]. While the direction of change is too complicated to predict, plausible mechanisms for climate change to alter reservoir host ecology in the Canadian North include, but are not limited to, altered overwinter survival with deeper snowpacks, increased competition from invasive species, increased length of feeding and breeding seasons, and increased pressure from other, more pathogenic diseases, and parasites that may alter survival and reservoir competence. Overall, it is likely that both SSHV and JCV are broad enough in their reservoir host preferences that they are resilient to climate-driven changes in mammalian diversity. The current high seroprevalence in caribou indicates that transmission is already widespread, and while this is not likely to be contributing to caribou declines, the effects of the viruses themselves are not known in free-ranging wildlife. In general, warming temperatures could be causing stress on host species such as caribou, making them more vulnerable to disease, including those that were not particularly pathogenic under normal circumstances, such as CSG viruses (Bradley et al., 2018).

## 6. Public Health Messaging

Frontline medical providers need to consider arboviruses, and specifically CSG viruses, as a differential diagnosis for neurological disease and order appropriate serological and molecular testing. Serosurveys in Canada have only been conducted in isolated communities and regions, and more broad sampling (perhaps through blood banks) is needed to determine the level of exposure in the general population. Snowshoe Hare virus and JCV are transmitted by mosquitoes, which means that hunters and trappers in northern communities likely do not acquire the viruses while working directly with wildlife or harvested carcasses. However, safe carcass handling, including the use of hand washing and gloves, remains recommended [8]. Even so, the risk of exposure increases with time spent outdoors [92]. As mosquitoes are the primary means of transmission of these viruses, transmission to mammals is likely limited to the summer months when mosquitoes are active in the North. Mitigation strategies targeting human exposure include public health messaging promoting the use of effective insect repellents and long clothing to prevent bites. Reducing the interaction between wildlife and mosquitoes is nearly impossible and reducing the population density of mosquitoes via insecticides, larvicides or the Sterile Insect Technique (releasing large numbers of sterilized males) is currently impractical due to a lack of infrastructure and a vast geographic area in the north. The use of any of these techniques would also need to be accompanied by a detailed risk assessment for potential non-target impacts. The benefits of traditional and cultural activities, including outdoor activities during peak mosquito activity during the summer, likely outweigh the risks of infection, though preventative measures can be used to reduce the potential for insect bites.
